# Treatment of a descending thoracic mycotic aneurysm secondary to disseminated aspergillosis infection with thoracic endovascular aortic repair

**DOI:** 10.1016/j.jvscit.2022.04.009

**Published:** 2022-05-12

**Authors:** Krystina N. Choinski, Joshua D. Harris, Peter V. Cooke, Rami O. Tadros

**Affiliations:** Division of Vascular Surgery, Department of Surgery, The Icahn School of Medicine at Mount Sinai, New York, NY

**Keywords:** Disseminated aspergillosis, Mycotic thoracic aortic aneurysm, Thoracic endovascular aneurysm repair

## Abstract

Mycotic aortic aneurysms are a rare and potentially fatal aortic pathology. Advancements in vascular technology have allowed endovascular repair to be a durable and less invasive option for the treatment of mycotic aortic aneurysms. We have presented the case of a 51-year-old man with a mycotic aneurysm of the descending thoracic aorta secondary to chronic, disseminated aspergillosis infection after liver transplantation. The aneurysm was successfully treated with thoracic aortic stent graft deployment. No perioperative complications occurred, and follow-up computed tomography angiography showed no signs of an endoleak. The patient will continue with lifelong antifungal therapy and close follow-up with vascular surgery.

Although mycotic aneurysms represent 0.7% to 2.6% of all aortic aneurysms, the risk to patients is high. The mortality rate of a rupture can be nearly 100%, even with intervention. Historically, these aneurysms were treated through open surgery with debridement and bypass, either in situ or extra-anatomic.^1^^,^^2^ Thoracic endovascular aortic repair (TEVAR) offers a lifesaving, less morbid alternative, although patients will require lifelong antibiotic therapy.^3^ We have presented a rare case of a descending thoracic mycotic aortic aneurysm (MAA) secondary to aspergillosis that was successfully treated with TEVAR. The patient provided written informed consent for the report of his case details and imaging studies.

## Case report

A 51-year-old man with alcoholic hepatitis and orthotopic liver transplantation complicated by multi-organismal infections presented with chronic lower back pain. His post-transplant infections included cytomegalovirus, enterococcal abdominal hematoma requiring a 3-month hospitalization, and disseminated aspergillosis (with lifelong isavuconazole therapy).

Prior magnetic resonance imaging of the spine had displayed osteomyelitis, likely secondary to the aspergillosis, at levels T12-L1. Given this persistent back pain, a computed tomography (CT) scan without contrast enhancement was obtained. The CT scan displayed a nodular density in the left lower lobe adjacent to the descending thoracic aorta that had increased in size from 5 months previously and was concerning for an enlarging pulmonary nodule vs an MAA.

**Fig 1:**
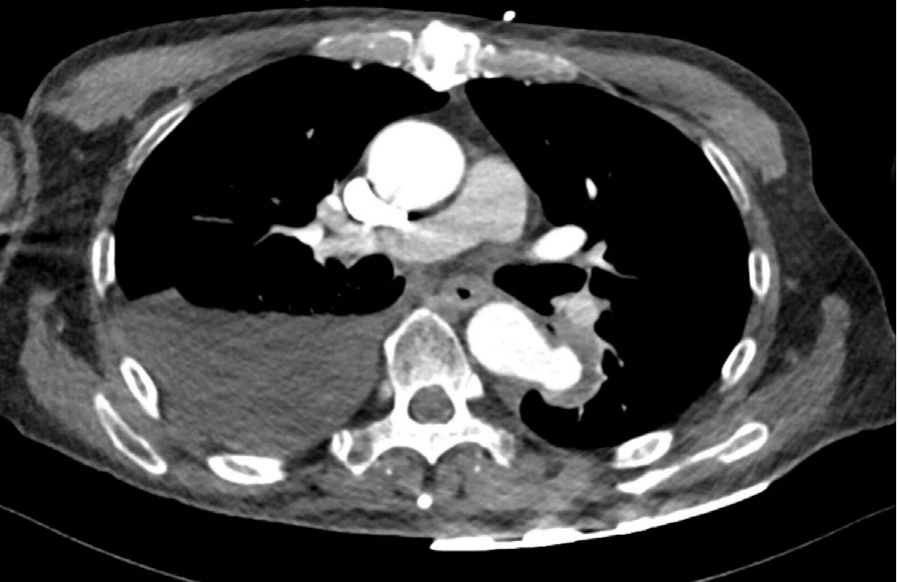
Computed tomography angiography (CTA) displaying axial view of a saccular, 2.4 × 2.9 × 2.5 cm, mycotic aneurysm of the descending thoracic aorta. The aneurysm neck was ∼1.4 cm, and an 8-mm outer portion was thrombosed.

**Fig 2:**
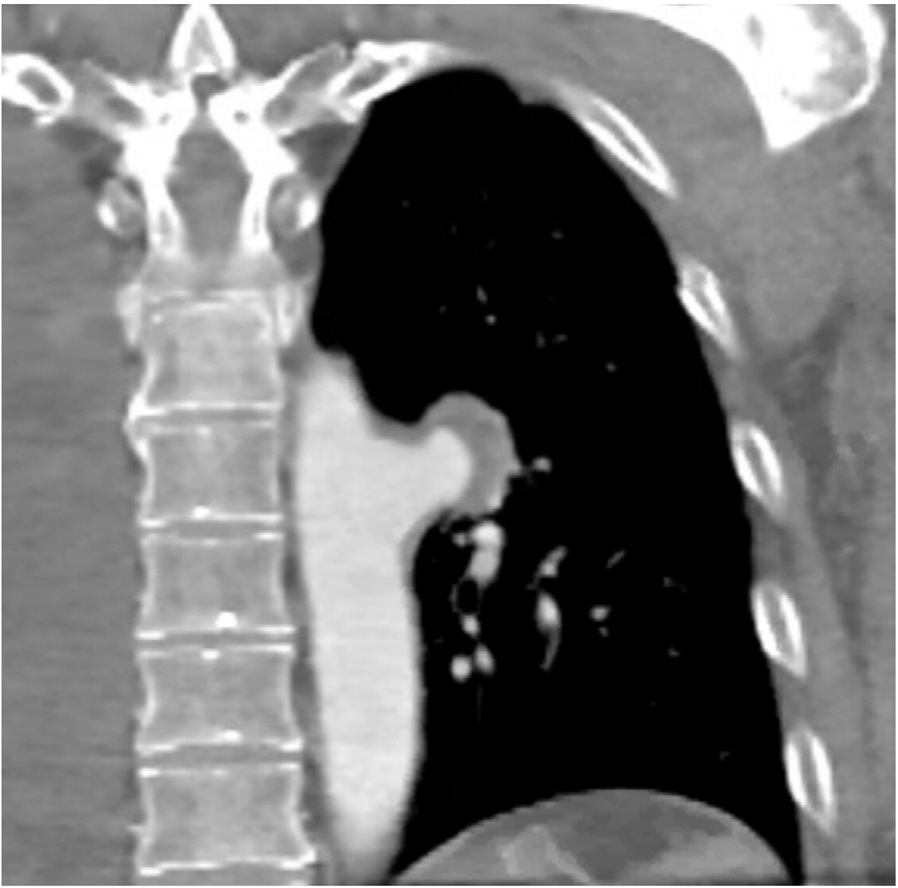
Computed tomography angiography (CTA) displaying coronal view of a saccular, 2.4 × 2.9 × 2.5 cm, mycotic aneurysm of the descending thoracic aorta.

**Fig 3:**
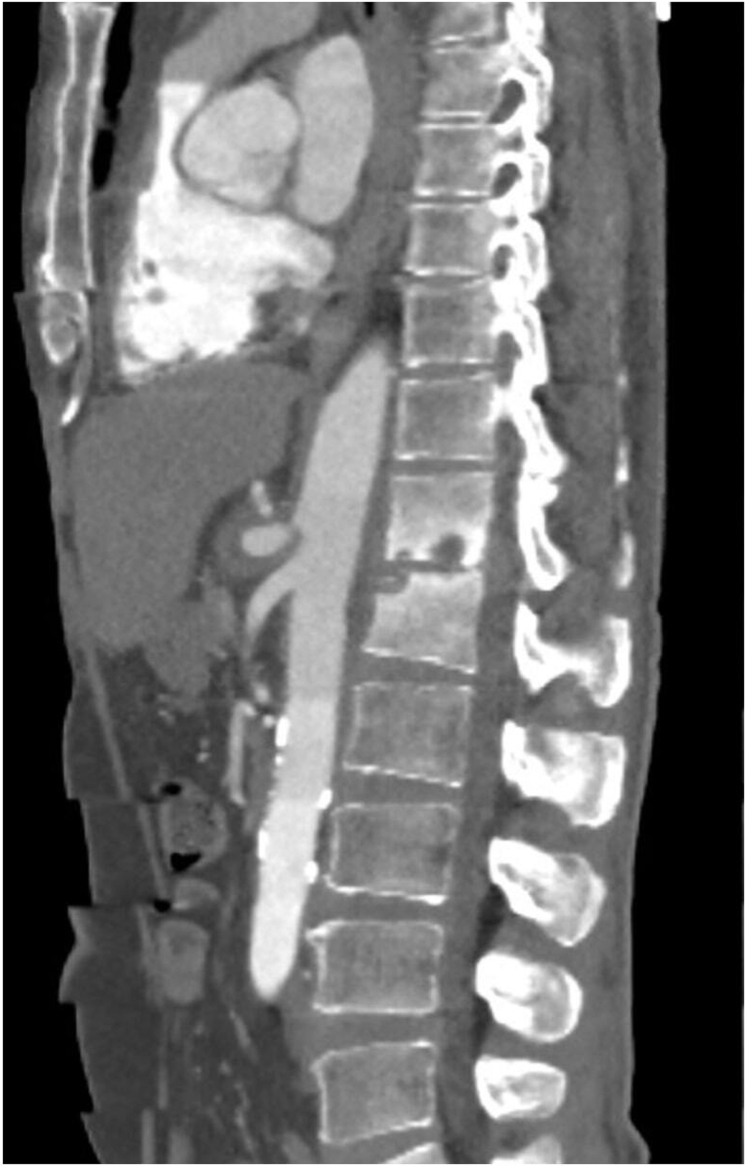
Computed tomography angiography (CTA) displaying sagittal view of focal aneurysmal dilatation of the proximal celiac trunk, ≤1.1 cm, with subsequent total occlusion of the celiac artery ∼2 cm from its ostium.

The patient was admitted, and amphotericin was started. Vascular surgery was consulted, and the patient underwent CT angiography (CTA), which revealed a saccular, wide-necked aneurysm measuring 2.4 × 2.9 × 2.5 cm at the descending thoracic aorta, distal to the left pulmonary artery (Fig 1). Approximately 8 mm of the aneurysm appeared thrombosed. The aneurysmal neck was 1.4 cm (Fig 2). A bovine arch, aneurysmal dilatation of the proximal celiac trunk ≤1.1 cm, and total occlusion of the celiac artery 2 cm from its origin (chronic; noted on a prior scan) were present (Fig 3). All other visceral vessels were patent, and the celiac branches were perfused via collateral vessels.

The decision was made to pursue TEVAR. Given the patient’s major abdominal surgery, prolonged hospital course, intra-abdominal infection, and malnourishment, he had an extremely high risk of complications with open surgery. Endovascular repair would decrease procedural morbidity, with the plan to continue lifelong antifungal therapy. We informed the patient that TEVAR would be safer given his clinical condition but noted the need for close monitoring, possible reintervention, and the risk of further aneurysmal degeneration or rupture.

**Fig 4:**
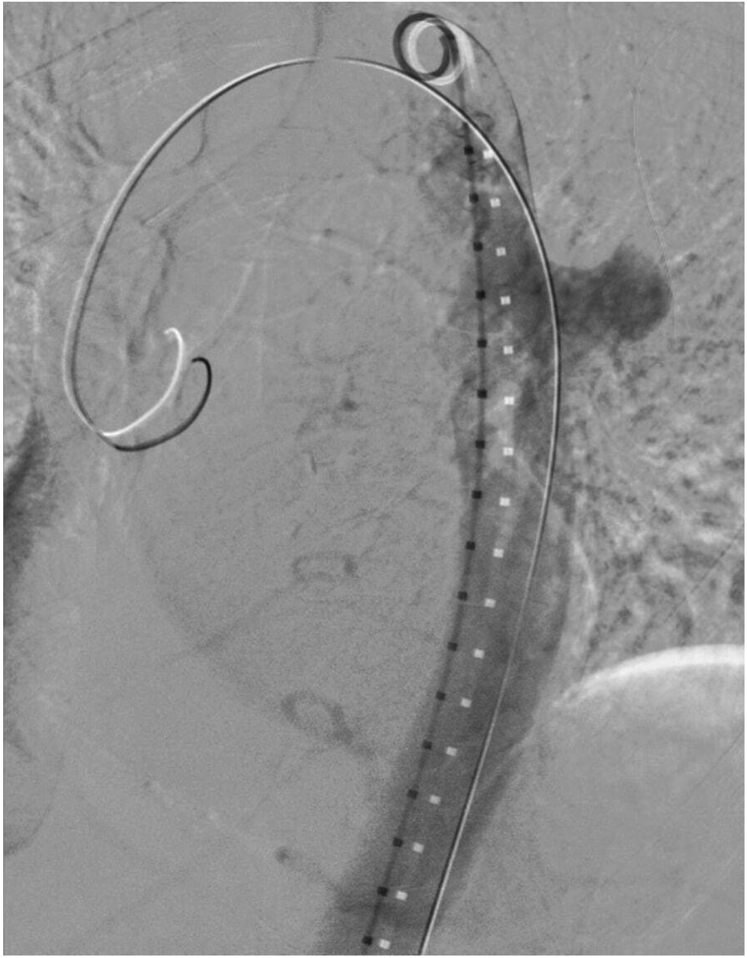
Aortogram displaying a saccular mycotic aneurysm off the descending thoracic aorta.

**Fig 5:**
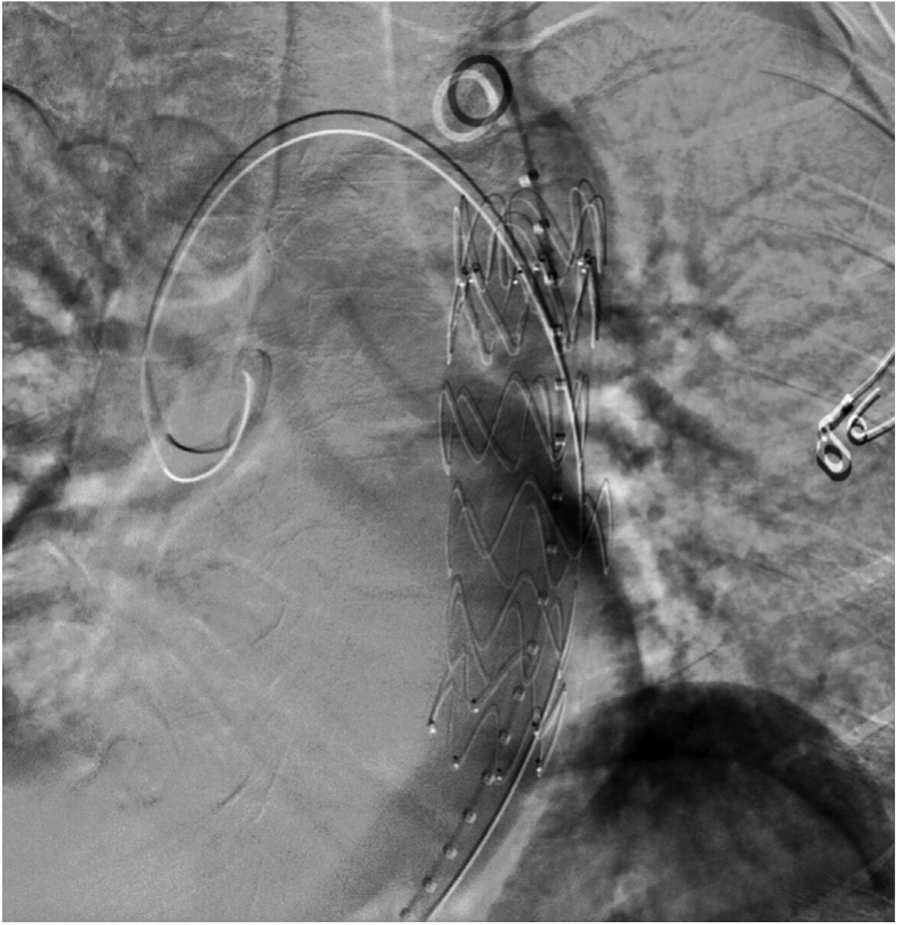
Completion aortogram displaying successful thoracic endovascular graft deployment and exclusion of the saccular mycotic aneurysm of the descending thoracic aorta.

Bilateral common femoral access was achieved. A 5F sheath was placed in the left femoral artery and the double preclose technique was performed in the right femoral artery for an 8F sheath. A diagnostic catheter was placed just distal to the aortic arch, and an aortogram identified the MAA (Fig 4). The Zenith alpha thoracic endovascular graft D 32 × 109 device (Cook Medical Inc, Bloomington, IN) was deployed with two stent forms above and below the aneurysm. No endoleak was found on the completion aortogram (Fig 5). The left common femoral arteriotomy was closed via the perclose technique. No perioperative complications had occurred.

**Fig 6:**
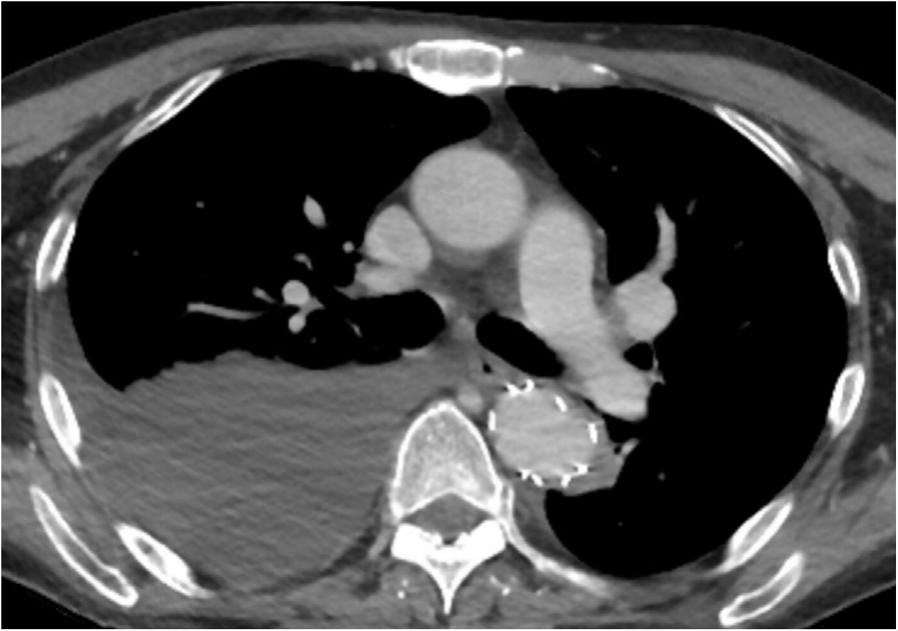
Computed tomography angiography (CTA) displaying axial view after thoracic endograft deployment showing successful exclusion of the saccular mycotic aneurysm and no evidence of an endoleak.

Postoperatively, the patient remained hospitalized for an infectious workup. Magnetic resonance imaging of the spine showed T12-L1 osteomyelitis and phlegmon. A bone biopsy was performed, with all culture findings negative. The patient’s back pain improved, and he was discharged with lifelong posaconazole therapy. His posaconazole and tacrolimus dosages have been closely managed by both transplant and infectious disease providers. He returned to vascular surgery clinic at 2 months postoperatively. CTA at 2 months postoperatively showed the absence of any endoleak (Fig 6).

No complications related to the aortic repair occurred. The patient was admitted 4 months after the procedure for decompensated heart failure with severe mitral regurgitation and valvular vegetations. He underwent mitral valve and tricuspid valve repair. His postoperative course was uneventful, and he continued taking posaconazole for chronic fungemia. Repeat imaging studies at that time displayed regression of the aneurysm sac and no evidence of an endoleak. CTA has been planned for the 1-year follow-up examination to monitor the aortic repair.

## Discussion

Aspergillus is the most common fungal infection in humans, causing a variety diseases.^4^ Noninvasive infections include allergic sinusitis and allergic bronchopulmonary aspergillosis. The respiratory tract is a common site of invasive infections; however, any organ can become infected.^5^ *Aspergillus* MAAs are rare, with a high mortality,^6^^,^^7^ occurring in immunocompromised patients with granulocytopenia.^7^, ^8^, ^9^ *Aspergillus* MAAs occur after cardiac surgery, with infected valves and sutures,^10^, ^11^, ^12^, ^13^ and can be secondary to pyelonephritis or endocarditis.^14^, ^15^, ^16^ Fungal MAAs can affect the aortic arch and the ascending, thoracic, and abdominal aortas.^7^^,^^12^^,^^15^, ^16^, ^17^, ^18^ *Aspergillus* infection will occur in 1% to 9% of liver transplant patients, with mortality >20%. The current guidelines have recommended prophylactic antifungal agents for high-risk transplant patients.^19^ The primary treatment has been voriconazole, with liposomal amphotericin B an alternative.^20^ Temporarily reducing or eliminating immunosuppressive agents has been recommended when feasible.^20^

Open surgical repair of MAAs has resulted in high mortality of ∼28%.^6^^,^^21^^,^^22^ Despite successful surgery, the overall prognosis has been poor.^23^ In contrast, a mortality benefit exists for endovascular repair.^24^ A systematic review of 983 MAA patients showed that EVAR resulted in improved short-term mortality without increased infection-related complications.^21^ Compared with the review cohort, our patient was younger, with a thoracic aneurysm vs abdominal and *Aspergillus* vs bacterial infection. Although reports of the repair of *Aspergillus* MAAs have been sparse, prior studies have suggested that EVAR and TEVAR are effective.^6^^,^^25^ To the best of our knowledge, the present report is the first to describe TEVAR repair for *Aspergillus* MAA.

Semba et al^26^ first described endovascular repair of mycotic descending aortic aneurysms. No perioperative mortality or complications had resulted from the bacteremia.^26^ The complications associated with TEVAR for MAAs include perioperative rupture, stent migration, and type I endoleaks.^27^ In a series of four ruptured and one intact thoracic MAA, TEVAR resulted in one perioperative death and one type II endoleak during a 30.5-month follow-up period.^28^ We have uniquely reported the management of fungal, in contrast to previously described bacterial, MAAs.

At the last follow-up, we had encountered no complications at 8 months after TEVAR. The nonrupture status of the aneurysm allowed for case planning and precise graft deployment, permitting operative success and the lack of complications. We witnessed almost complete aneurysm sac regression, likely owing to the prolonged antifungal course both pre- and postoperatively.

Survival after TEVAR for MAA has varied.^21^^,^^26^^,^^27^ The 30-day mortality, due to sepsis or massive bleeding, was 10.4%.^29^ The late causes of mortality include cardiac- and graft-related bleeding complications. The 12-month survival rate of the healed group was 94.0% ± 4.0% vs 39.0% ± 17.0% for the persistently infected group. The 2-year survival rate was 82.2% ± 5.8%.^29^ The significant predictors of adverse outcomes included age >65 years, aneurysm rupture, fever before surgery, and non–*Salmonella*-positive cultures. The major complications of TEVAR for MAAs include infection-related complications and late death secondary to rupture.^21^^,^^27^ Given our patient’s disseminated aspergillosis, close surveillance is essential. We acknowledge that open repair of MAAs remains the reference standard with intraoperative debridement and in situ prosthetic reconstruction, followed by lifelong antibiotic suppression therapy.^30^ However, given our patient’s deconditioned status after his liver transplantation and prolonged hospitalization, we believed TEVAR would allow for the best outcome. Additionally, the use of TEVAR could be a bridge to open aortic repair, if required. The patient’s functional status has largely improved postoperatively, such that he would more likely tolerate an open intervention.

Our patient will require a lifelong antifungal regimen. Despite this therapy, the risk of complications is high given the infected field. Close postoperative surveillance and annual CT imaging studies will be key for monitoring the patient’s aortic repair. If an endoleak develops or graft migration occurs, further intervention remains an option.

## Conclusions

The present case represents successful TEVAR of a rare mycotic descending thoracic aortic aneurysm secondary to chronic, disseminated aspergillosis.
